# Survival among Patients with HIV Infection and Smear-Negative Pulmonary Tuberculosis - United States, 1993–2006

**DOI:** 10.1371/journal.pone.0047855

**Published:** 2012-10-23

**Authors:** J. Sean Cavanaugh, N. Sarita Shah, Kevin P. Cain, Carla A. Winston

**Affiliations:** 1 Epidemic Intelligence Service Officer, Division of Tuberculosis Elimination, U. S. Centers for Disease Control and Prevention, Atlanta, Georgia, United States of America; 2 Division of Tuberculosis Elimination, U.S. Centers for Disease Control and Prevention, Atlanta, Georgia, United States of America; 3 Department of Medicine, Albert Einstein College of Medicine, Bronx, New York, United States of America; San Francisco General Hospital, University of California San Francisco, United States of America

## Abstract

**Background:**

In patients with HIV and tuberculosis (TB) in resource-constrained settings, smear-negative disease has been associated with higher mortality than smear-positive disease. Higher reported mortality may be due to misdiagnosis, diagnostic delays, or because smear-negative disease indicates more advanced immune suppression.

**Methods:**

We analyzed culture-confirmed, pulmonary TB among patients with TB and HIV in the United States from 1993–2008 to calculate prevalence ratios (PRs) for smear-negative disease by demographic and clinical characteristics. Allowing two years for treatment outcome to be reported, we determined hazard ratios (HRs) for survival by smear status, adjusted for significant covariates on patients before 2006.

**Results:**

Among 16,710 cases with sputum smear results, 6,739 (39%) were sputum smear-negative and 9,971 (58%) were sputum smear-positive. The prevalence of smear-negative disease was lower in male patients (PR: 0.89, 95% confidence interval [CI]: 0.86–0.93) and in those who were homeless (PR: 0.92, CI: 0.87–0.97) or used alcohol excessively (PR: 0.91, CI: 0.87–0.95), and higher in persons diagnosed while incarcerated (PR: 1.20, CI: 1.13–1.27). Patients with smear-negative disease had better survival compared to patients with smear-positive disease, both before (HR: 0.82, CI: 0.75–0.90) and after (HR: 0.81, CI: 0.71–0.92) the introduction of combination anti-retroviral therapy.

**Conclusions:**

In the United States, smear-negative pulmonary TB in patients with HIV was not associated with higher mortality, in contrast to what has been documented in high TB burden settings. Smear-negative TB can be routinely and definitively diagnosed in the United States, whereas high-burden countries often rely solely on AFB-smear microscopy. This difference could contribute to diagnostic and treatment delays in high-burden countries, possibly resulting in higher mortality.

## Introduction

Sputum smear examination of acid-fast bacilli (AFB) is the cornerstone of tuberculosis (TB) diagnosis worldwide. Before the HIV epidemic, AFB smear-negative TB was diagnosed in a smaller proportion of TB patients than it is today and was regarded as earlier or less severe disease [1]. Patients diagnosed with smear-negative TB are less infectious and, prior to the HIV epidemic, had lower morbidity and mortality than smear-positive TB [2]–[5]; for these reasons, smear-negative TB disease has been a lower priority for TB control efforts. In resource-limited countries, however, the proportion of pulmonary TB disease that is smear-negative has increased over the past two decades, a shift largely attributed to the growing HIV epidemic [6], [7]. Moreover, in countries with a high prevalence of HIV, patients diagnosed with smear-negative TB have been shown to have higher mortality than patients with smear-positive TB [8]–[12], raising new questions and concerns about this form of TB disease.

A number of explanations for the increased mortality associated with smear-negative TB in resource-limited, high HIV prevalence countries have been proposed. Some researchers suggest that smear-negative disease in HIV-infected patients may indicate more advanced immune suppression, which may be mediating the observed differences in survival [1], [8]–[10]. Laboratory confirmation is rarely performed in countries with high HIV prevalence, and others conjecture that diagnostic inaccuracy and incorrect therapy are important reasons for the high mortality [8], [11], [12]. In addition, practical difficulties diagnosing smear-negative TB – such as the need for additional smears, chest radiographs, or trials of antibiotics – lead to delays in appropriate treatment, which have been clearly associated with poor outcomes [13]–[15]. Finally, some countries did not always include rifampicin in the continuation phase for smear-negative disease, which may explain the observed higher mortality in patients with HIV infection [8].

Analyses of the association between sputum AFB smear result and mortality in patients with HIV and TB that assess these hypotheses would be helpful in designing interventions to improve outcomes. In the United States, TB is diagnosed by means of extensive laboratory testing, which includes microscopy, nucleic acid amplification and specimen culture [16]. The widespread use of testing to confirm pulmonary TB, along with a well-established national TB surveillance system with over 15 years of data, provide a unique opportunity to investigate the association between AFB smear result and treatment outcomes in the United States. Additionally, because the diagnostic process is different in the United States than in resource-limited settings, comparing mortality of smear-negative TB patients between these two settings may help refine hypotheses for the observed increase in mortality seen in resource-limited settings. We used the U.S. National TB Surveillance System (NTSS) to investigate clinical features associated with AFB smear status in TB patients infected with HIV and analyzed the effect of AFB smear status on survival.

## Methods

### Ethical Considerations

Data were collected and analyzed as part of routine public health surveillance and not human subjects research, and therefore institutional review board approval was not required.

### Study Population

In the United States since 1993, all states and affiliated jurisdictions have reported incident cases of TB to the Centers for Disease Control and Prevention (CDC) using a standardized case report form, the Report of Verified Case of Tuberculosis (RVCT) [17]. The RVCT includes socio-demographic, laboratory, and clinical information on TB patients as well as follow-up data on TB treatment outcomes. These data have been captured as well-defined variables [18].

We restricted our analysis to persons with positive HIV infection status reported on the RVCT, and included all sputum culture-confirmed cases of pulmonary TB, including those with both pulmonary and extrapulmonary involvement, that were reported by the District of Columbia and 48 of the 50 states. California did not report HIV status directly to the NTSS; HIV status of TB patients from California was determined by matching patients on the state-level TB and AIDS registries. HIV was missing for all California TB patients not matched to the state AIDS registry prior to 2005 and for all California TB cases from 2005–2008. Given the difference in reporting and potential for bias, we excluded TB patients from California from our analyses, and conducted separate analyses of California TB/AIDS patient mortality data. Additionally, Vermont stopped reporting HIV data to the CDC in 2006; because there were fewer than five Vermont patients with TB/HIV infection over the entire study period, we excluded patients from Vermont from analyses without examining state-specific mortality results.

For demographic and clinical analyses of cross-sectional data, we included all patients with a known smear status who were reported to the NTSS from January 1, 1993 until December 31, 2008. For survival analyses, we included only patients who were documented to be alive at diagnosis, initiated anti-TB therapy, and were reported to the CDC as a culture-confirmed case through December 31, 2006, to allow two years for reporting of TB treatment outcomes.

In order to account for the population effect of highly active anti-retroviral therapy (HAART) on survival, we conducted separate analyses for the time periods from 1993 to 1997, before HAART became widely available, and from 1998 to 2006, when patients with TB and HIV were routinely prescribed HAART.

There are no NTSS data collected about CD4 count, HIV viral load, or anti-retroviral use, so we were unable to assess the effect of these variables in our analysis.

### AFB Smear Definitions

A TB patient was sputum smear-positive if any result of a microscopic examination of a sputum smear (expectorated or induced) was positive for acid-fast bacilli (AFB). This excludes pulmonary secretions obtained by tracheal aspirations, bronchoscopy procedures, or gastric aspiration. Sputum smear positivity grade is not available in the NTSS. A TB patient was sputum smear-negative if all results of microscopic examination of sputum smears were negative for AFB. As recommended, at least three samples of sputum are generally sent for microscopy in the United States [19].

### Statistical Analyses

To investigate features associated with AFB smear status, we compared frequency distributions of smear-negative TB with smear-positive disease across categorical demographic and clinical variables (gender, age group, race/ethnicity, nationality [defined as U.S.- or foreign-born], correctional facility at diagnosis, homelessness, excess alcohol use, injection drug use, previous TB diagnosis, tuberculin skin test [TST] result, and chest radiograph result), defined in the RVCT [18]. Associations are presented as prevalence ratios (PRs) along with 95% confidence intervals (CIs). Prevalence ratios can be interpreted as the proportion (or prevalence) of smear-negative disease among patients with a specific characteristic (e.g., male gender), divided by the proportion of smear-negative disease in a pre-determined reference group (e.g., female gender).

To assess potential treatment delays by AFB smear status, we compared the median time from collection date of the first sputum that grew *Mycobacterium tuberculosis* to the start date of TB therapy between smear-negative and smear-positive cases using a Wilcoxon two-sample test. The RVCT records the date that initial sputum was collected only for cases that had documented conversion from a positive to a negative culture. We were not able to analyze survival of patients who died before diagnosis was made because neither date of symptom onset nor reason for evaluation for TB disease are reported in the RVCT.

To investigate the relationship between AFB smear status and survival during TB treatment, we performed comparative survival analyses by smear status based on time from start of TB therapy to death (as a TB treatment outcome). To ensure uniform follow-up for treatment outcomes of all patients, we censored patients at 730 days and didn’t capture specific treatment outcomes if they were recorded more than two years after the start of therapy. Patients with outcomes other than death were censored based on either date of stopping TB therapy or last known follow-up. Overall survival distributions for both groups were estimated using the Kaplan-Meier method and compared via log-rank test statistics. We fit a Cox multivariable proportional hazards model after testing the proportional hazards assumption for each variable. We identified demographic and behavioral variables that were either epidemiologically relevant, or were significantly associated with mortality in bivariate analysis (p<0.01) and adjusted for them; these variables included age group, race, nationality, incarceration at diagnosis, previous TB, as well as reported injection drug use (IDU), excessive alcohol use, and homelessness in the past year. We assessed variables for effect modification and, given the large sample size, dropped from the model all variables that did not meet a significance level of p<0.01. All statistical analyses were performed using SAS, version 9.2 (Cary, NC).

## Results

### Case Estimates and Trends

**Figure 1:**
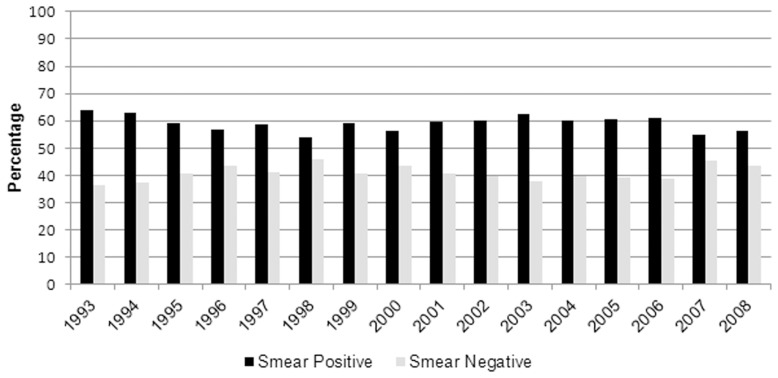
Sputum smear status among patients with HIV and culture-confirmed TB among those with reported sputum smear, United States, 1993–2008. Black bars indicate the percentage of patients with reported sputum smear result who were smear-positive. Gray bars indicate the percentage of patients with who were smear-negative.

From 1993 through 2008, there were 17,089 culture-confirmed patients with pulmonary TB and HIV infection, 16,710 of whom had a sputum smear result reported. Of those, 6,739 (40%) were sputum smear-negative and 9,971 (60%) were sputum smear-positive. The annual proportion of TB cases that were smear-negative varied from 36% to 45%; however, there was no apparent linear trend (Figure 1).

### Associated Characteristics of Smear-Negative TB

Of the 6,739 patients with HIV infection and smear-negative TB, the majority were male (n = 4,922 or 73%), black (n = 4,574 or 68%), aged 25–44 (n = 4,570 or 68%) and U.S.-born (n = 5,149 or 77%) (Table 1). Smear-negative TB was more prevalent among persons in correctional facilities at the time of TB diagnosis (PR: 1.20, CI: 1.13–1.27) and less prevalent among patients who were homeless (PR: 0.92, CI: 0.87–0.97) (Table 1). The prevalence of smear-negative disease was lower among those who used alcohol excessively compared to those who did not (PR: 0.91, CI: 0.87–0.95), but did not differ among patients by injection drug use or history of previous TB diagnosis (Table 2).

**Table 1:** Demographic Characteristics of Patients with HIV and Culture-Confirmed, Pulmonary TB, by Sputum AFB Smear Result, United States, 1993–2008.

Characteristic	Smear-Negativen (%)*	Smear-Positiven (%)*	Prevalence Ratio of Smear-Negative Disease (95% Conf. Int.)
**Total**	**6,739**	**9,971**	
**Gender**			
Male	4,922 (39)	7,642 (61)	0.89 (0.86–0.93)
Female	1,817 (44)	2,329 (56)	Referent
**Age in years** †			
0–14	17 (53)	15 (47)	1.32 (0.95–1.82)
15–24	192 (39)	296 (61)	0.97 (0.87–1.09)
25–44	4,569 (40)	6,750 (60)	Referent
45–64	1,846 (40)	2,723 (60)	1.00 (0.96–1.04)
65+	114 (38)	187 (62)	0.94 (0.81–1.09)
**Race/ethnicity** §			
Hispanic	1,260 (39)	2,010 (61)	0.95 (0.88–1.01)
American Indian/Alaska Native	16 (33)	32 (67)	0.82 (0.55–1.23)
Asian	89 (36)	161 (64)	0.87 (0.73–1.04)
Black or African-American	4,574 (41)	6,599 (59)	1.01 (0.95–1.07)
Native Hawaiian/Pacific Islander	0 (0)	4 (100)	n/a
White	776 (41)	1,131 (59)	Referent
**Nationality**			
U.S.-born	5,148 (40)	7,623 (60)	Referent
Foreign-born	1,553 (40)	2,297 (60)	1.00 (0.96–1.05)
Unknown	38 (43)	51 (57)	1.06 (0.83–1.35)
**Correctional facility resident (at time of TB diagnosis)** **			
Yes	627 (48)	688 (52)	1.20 (1.13–1.27)
No	5,876 (40)	8,893 (60)	Referent
Unknown/Missing	104 (36)	188 (64)	0.90 (0.77–1.05)
**Homeless (within past year)** **			
Yes	883 (37)	1,489 (63)	0.92 (0.87–0.97)
No	4,901 (41)	7,144 (59)	Referent
Unknown/Missing	823 (42)	1,136 (58)	1.03 (0.98–1.09)

* Percentages are the proportion with a specific smear status among all patients with the characteristic who had a known smear status.

† One smear-negative case had an unknown age and is not included.

§ There were 6 smear-positive cases and 2 smear-negative cases designated as multiple race and 28 smear-positive and 22 smear-negative cases designated as unknown race, these were not included.

** Among patients aged 15–64 (6,607 smear-negative, 9,769 smear-positive).

**Table 2:** Behavioral and Clinical Characteristics of Patients with HIV-Infection and Culture-Confirmed Pulmonary TB, by Sputum Smear Result, United States, 1993–2008.

Characteristic	Smear-Negativen (%)*	Smear-Positiven (%)*	Prevalence Ratio of Smear-Negative Disease(95% Conf. Int.)
**Excess alcohol use (within past year)** †			
Yes	1,410 (38)	2,329 (62)	0.91 (0.87–0.95)
No	3,936 (41)	5,563 (59)	Referent
Unknown/Missing	1,255 (40)	1,877 (60)	0.97 (0.92–1.02)
**Injection drug use (within past year)** †			
Yes	804 (41)	1,155 (59)	1.01 (0.96–1.07)
No	4,548 (40)	6,685 (60)	Referent
Unknown/Missing	1,255 (39)	1,929 (61)	0.97 (0.93–1.02)
**Previous TB diagnosis** §			
Yes	321 (38)	523 (62)	0.94 (0.86–1.03)
No	6,358 (40)	9,361 (60)	Referent
Unknown/Missing	60 (41)	87 (59)	1.01 (0.83–1.23)
**Tuberculin skin test (TST) result** §			
Positive	2,467 (46)	2,911 (54)	Referent
Negative	1,537 (38)	2,539 (62)	0.82 (0.78–0.86)
Unknown	2,735 (38)	4,521 (62)	0.82 (0.79–0.86)
**Chest Radiograph** **			
Normal	1,051 (60)	707 (40)	1.62 (1.55–1.70)
Abnormal – consistent with TB	4,881 (36)	8,375 (64)	Referent
Abnormal – not consistent with TB	287 (55)	237 (45)	1.49 (1.37–1.61)
Abnormal – unknown result	262 (42)	366 (58)	1.13 (1.03–1.25)

* Percentages are the proportion with a specific smear status among all patients with the characteristic who had a known smear status.

† Among patients aged 15–64 (6,607 smear-negative, 9,769 smear-positive).

§ Among all patients (6,739 smear-negative, 9,971 smear-positive).

** Among patients with a reported chest radiograph result (6,481 smear-negative, 9,685 smear-positive).

### Time to Initiation of Therapy

Among patients who were alive at diagnosis and had available date of initial sputum collection, the median number of days between the collection of the first sputum specimen that grew *M. tuberculosis* to the start date of therapy was 1 (75^th^ percentile = 5, 25^th^ percentile = 0; IQR = 5) for the 6,698 smear-positive patients and 12 (75^th^ percentile = 35, 25^th^ percentile = 0; IQR = 35) for the 4,342 smear-negative patients, indicating a significant delay in treatment initiation (p<0.0001).

### Survival Analyses

**Figure 2:**
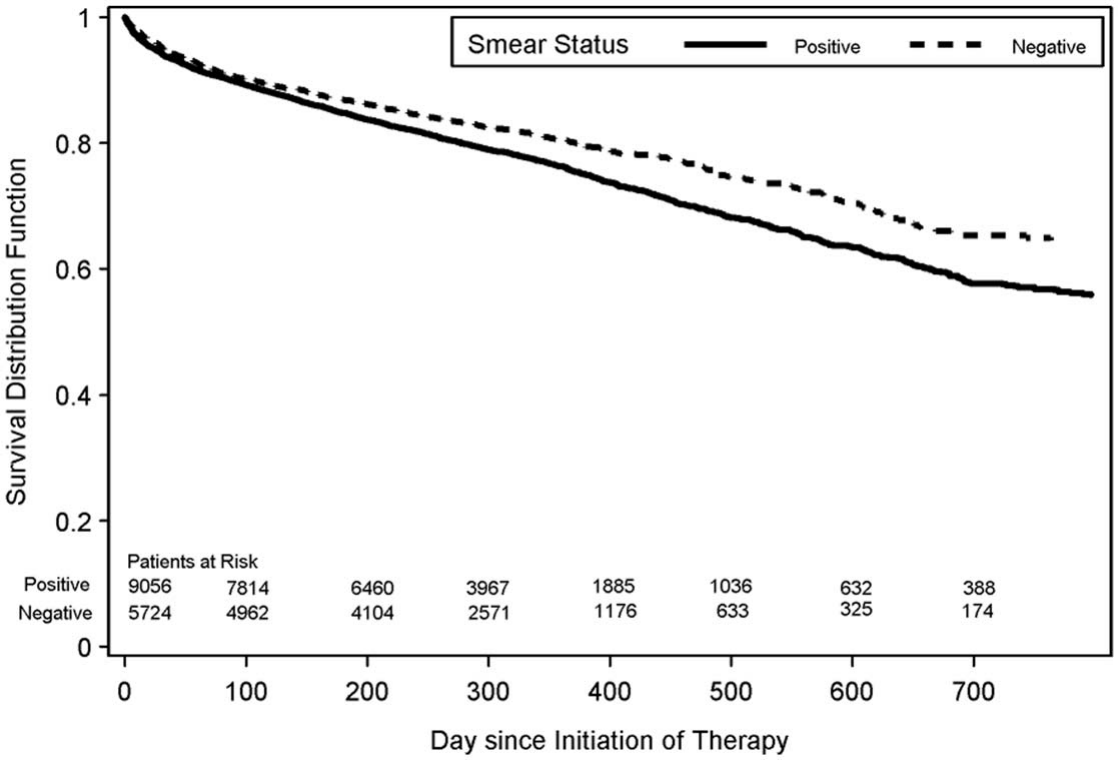
Survival Curves for patients with HIV-infection and culture-confirmed pulmonary TB, by sputum smear result, United States, 1993–2006. Solid line indicates the Kaplan-Meier survival curve for smear-positive patients. Dotted line indicates the Kaplan-Meier survival curve for smear-negative patients.

Patients with HIV and smear-negative TB had significantly better survival during TB treatment than patients with HIV and smear-positive TB (log rank statistic = 36.1, p<0.0001) (Figure 2). This difference persisted in sub-group analysis of TB/HIV patients diagnosed before the widespread use of HAART (1993–1997) (log rank statistic = 22.7, p<0.0001) and after HAART became widely available (1998–2006) (log rank statistic = 10.9, p = 0.0009).

In the multivariate Cox proportional hazards model, smear-negative TB/HIV patients had a 19% lower risk of death during TB treatment, even after adjusting for gender, age group, race/ethnicity, nationality, correctional institution residence at time of diagnosis, IDU, and alcohol; the hazard ratio was similar for TB/HIV patients diagnosed from 1993–1997 and for patients diagnosed from 1998–2006 (Table 3). When we conducted separate analysis of California TB patients who matched to their state AIDS registry, patients with smear-negative TB had an even lower risk for death than patients with smear-positive disease (adj HR 0.67, CI: 0.53–0.84 for patients diagnosed from 1993–1997; adj HR 0.79, CI: 0.49–1.28 for patients diagnosed from 1998–2006).

**Table 3:** Hazard Ratios for Death among Patients with HIV-infection and Culture-Confirmed Pulmonary TB Alive at Diagnosis and Initiated Therapy, United States, 1993–2006.

Population*	Died	Completed Therapy†	Adj. HazardRatio§ (95% Conf. Int.)
**HIV-infected, 1993–2006 (overall)**			
Smear-positive	2,212 (27)	6,107 (73)	Referent
Smear-negative	1,112 (22)	4,053 (78)	0.81 (0.76–0.88)
**HIV-infected, 1993–1997**			
Smear-positive	1,527 (33)	3,072 (67)	Referent
Smear-negative	756 (27)	2,026 (73)	0.82 (0.75–0.90)
**HIV-infected, 1998–2006**			
Smear-positive	685 (18)	3,035 (82)	Referent
Smear-negative	356 (15)	2,027 (85)	0.81 (0.71–0.92)

* Excluding patients reported after 2006 (536 smear-positive, 431 smear-negative), not documented as alive at diagnosis (191 smear-positive, 362 smear-negative), with no documented treatment (35 smear-positive, 112 smear-negative), or whose recorded outcome date preceded start of therapy or was missing (153 smear-positive, 110 smear-negative).

† Other outcomes not represented as a column in this table include patients who moved during treatment or were lost (650 smear-positive, 484 smear-negative), were uncooperative with therapy (52 smear-positive, 45 smear-negative), or whose outcomes were otherwise not known (35 smear-positive, 30 smear-negative),

§ Adjusted for gender, age group, race/ethnicity, nationality, incarceration, alcohol and drug use, and previous TB; after adjustment, homelessness in the previous year was the only examined covariate that was no longer statistically significant.

## Discussion

Our data demonstrate that, among U.S. patients with HIV, smear-negative TB patients who initiated treatment had significantly lower 2-year mortality than smear-positive patients. This was true even in the pre–HAART era, when mortality from HIV/AIDS was exceedingly high. The strengths of this study include the robustness of this population-based data, which has been estimated as >99% complete for capturing TB cases [20], the routine use of culture in the United States to confirm TB and the use of other diagnostic tests to identify opportunistic pulmonary infections other than TB.

The finding of lower mortality in patients with HIV treated for smear-negative TB in the United States is different from what has been reported in resource-limited settings [8]–[10], [21]–[23]. The significantly decreased hazard for death among smear-negative TB/HIV patients in our analysis suggests that those with smear-negative TB who were alive at the time diagnosis was established, overall, had earlier, less severe disease than patients with smear-positive TB.

Many studies have demonstrated that patients with HIV infection have higher rates of smear-negative TB [24]–[26], but it is unclear if this is due to screening, or misdiagnosis, or if immune suppression itself diminishes the bacillary density in sputum. In the United States, incarcerated persons usually undergo aggressive TB screening [27], and the increased prevalence of smear-negative disease among this population supports the effect of screening in identifying early stage disease. This, along with the higher prevalence of smear-negative disease in TB patients who have normal chest radiographs seen in this analysis, suggests that smear-negative disease may indeed be an early stage of TB in the United States, even among patients with HIV.

Smear status may correlate with degree of immune dysfunction, and it has been hypothesized that, because worsening immune suppression impairs pulmonary cavitation [28], [29] and cavitation correlates with smear-positivity [30], patients with HIV and TB who are smear-negative have more severe immune suppression [1], [6]. There is, however, little consistency among studies that have directly investigated the correlation between immune function, as measured by CD4 count, and smear status. Some studies have shown that sputum bacillary density appears to decrease with falling CD4 count [31], [32], but other studies have demonstrated that the more severely immune compromised patients seem to have higher rates of smear-positive TB disease [33]–[36]. The discrepant findings suggest that the correlation between immune functioning and sputum bacillary density may not be linear. While we were unable to directly account for immune suppression in our analysis, our findings do not support the hypothesis that among patients with HIV, smear-negative TB disease is associated with greater immune suppression than smear-positive TB disease.

Differences in the study populations themselves may also explain why our findings differ from those previously published from resource-limited settings with higher HIV prevalence. By including only TB cases with microbiologic confirmation of disease, we eliminated non-TB pulmonary diseases (e.g., *Pneumocystis jirovecii* pneumonia, fungal diseases, etc.,) that otherwise might be misdiagnosed as TB. In countries that are unable to perform routine sputum cultures, the diagnosis of pulmonary TB in a patient with negative sputum smears is based on clinical and radiologic findings. These findings, however, are often nonspecific in persons with HIV, especially those with smear-negative disease [26], [37]. In our results, for example, 1,051 (16%) of 6,481 smear-negative, culture-confirmed pulmonary TB patients with chest radiograph reports had films that were interpreted as normal. In studies that did not use culture as a gold standard, it is likely that a substantial proportion of smear-negative TB patients was misdiagnosed and inappropriately treated, thus leading to higher mortality.

It is probable that patients with smear-negative TB in resource-limited countries have even more considerable diagnostic and treatment delays than smear-negative TB/HIV patients in the United States. Because smear microscopy is frequently the sole diagnostic laboratory test available, sputum examinations are usually repeated multiple times for TB patients whose initial sputum smears are negative for AFB, causing significant delays in diagnosis and treatment [13], [14]. Indeed, current international guidelines suggest that, in the absence of mycobacterial culture, a minimum of at least two sputum specimens negative for AFB, radiographic abnormalities consistent with active TB, laboratory confirmation of HIV infection or strong clinical evidence of HIV infection, and a decision by a clinician to treat with anti-TB medications should be documented to categorize a patient as a smear-negative TB case [38]. These delays have deleterious consequences for both the individual and public health [15], [39]. Delays in TB diagnosis among patients with HIV are particularly dangerous for the individual since HIV infection in patients with TB, regardless of smear status, is associated with high early mortality [7], [40]. In our analysis, time to initiation of therapy was significantly longer for surviving patients with smear-negative TB than for patients with smear-positive disease. These diagnostic and treatment delays, and their effect on survival, are likely to be smaller in the United States, where ancillary studies (such as sputum culture and nucleic acid amplification) are often submitted at the same time that smears are collected, than in countries where ancillary tests may not be readily available [39], [41].

Finally, it is also possible that differences in treatment contributed to the different findings. In some resource-limited countries, patients with smear-negative TB were not always treated with rifampicin throughout their treatment regimens [1]. Moreover rifampicin, because of its spectrum of activity, may treat some infections other than TB [7], [42]. The RVCT does not collect TB case management details, so we were not able to examine the effect of changes in TB treatment, or adherence to treatment, over time.

This study must be interpreted with the following considerations. Fluorescent microscopy has been increasingly used in the United States over the time period studied, and it is more sensitive for detection than microscopy used in most high TB burden settings [43]. Thus, findings from smear-negative TB patients in this study may differ from those among patients designated as sputum smear-negative based on less sensitive microscopy methods. A difference in the sensitivity of diagnostic technique, however, might explain a change in the size of the hazard rate between smear-negative TB and smear-positive TB, but would not account for a reversal. We were not able to examine patient-level effects of HAART since HIV treatment data is not reported on the RVCT; yet ecologic analyses did not suggest differences in the relationship between smear-negative TB disease and mortality in the pre- and post-HAART data. Importantly, we were unable to include patients who died before diagnosis was established, so we cannot make conclusions about all persons with smear-negative TB disease, and these findings are directly relevant only to patients who are alive at diagnosis and initiate therapy.

In the United States, mortality in patients with HIV infection was not higher among patients with smear-negative TB disease, as compared to those with smear-positive disease, and it is reasonable to conclude that smear-negative TB in patients who initiate treatment appears to represent an earlier or less severe form of TB disease. The difference between these findings and those from resource-limited settings is likely because diagnostics are better in this resource-rich setting. Accurate and timely TB diagnosis remains of paramount importance worldwide, especially as the global HIV epidemic confounds clinical presentation and worsens disease outcomes, and the findings of this analysis argue for the need for improved, affordable diagnostic tests in high-burden, resource-limited countries. In addition, greater efforts to identify TB cases early through active screening in high-risk groups are critical and must be combined with improved outreach to persons with limited healthcare access.
